# A Randomized Controlled Trial of the Efficacy of Systemic Enzymes and Probiotics in the Resolution of Post-COVID Fatigue

**DOI:** 10.3390/medicines8090047

**Published:** 2021-08-30

**Authors:** Abhijit Rathi, Swati B. Jadhav, Neha Shah

**Affiliations:** 1 Food Application and Development Laboratory, Advanced Enzymes Technologies Ltd., Louiswadi, Thane 400604, India; swati@advancedenzymes.com; 2 Pulmonary Fibrosis Now, Chino, CA 91710, USA; drnehashah772@gmail.com

**Keywords:** SARS-CoV-2 infection, COVID-19-induced fatigue, chalder fatigue scale, physical fatigue, mental fatigue, ImmunoSEB + ProbioSEB CSC3

## Abstract

Muscle fatigue and cognitive disturbances persist in patients after recovery from acute COVID-19 disease. However, there are no specific treatments for post-COVID fatigue. Objective: To evaluate the efficacy and safety of the health supplements ImmunoSEB (systemic enzyme complex) and ProbioSEB CSC3 (probiotic complex) in patients suffering from COVID-19 induced fatigue. A randomized, multicentric, double blind, placebo-controlled trial was conducted in 200 patients with a complaint of post-COVID fatigue. The test arm (*n* = 100) received the oral supplements for 14 days and the control arm (*n* = 100) received a placebo. Treatment efficacy was compared using the Chalder Fatigue scale (CFQ-11), at various time points from days 1 to 14. The supplemental treatment resulted in resolution of fatigue in a greater percentage of subjects in the test vs. the control arm (91% vs. 15%) on day 14. Subjects in the test arm showed a significantly greater reduction in total as well as physical and mental fatigue scores at all time points vs. the control arm. The supplements were well tolerated with no adverse events reported. This study demonstrates that a 14 days supplementation of ImmunoSEB + ProbioSEB CSC3 resolves post-COVID-19 fatigue and can improve patients’ functional status and quality of life.

## 1. Introduction

Coronavirus disease-19 (COVID-19), the disease caused by severe acute respiratory syndrome coronavirus 2 (SARS-CoV-2), is a worldwide pandemic afflicting a large population. Most infected people develop acute symptoms that last for 7–10 days. However, one or more symptoms (physical, cognitive and/or psychological) persist for weeks or even months in a substantial percentage of people [1]. Fatigue is the most persistent and debilitating symptom of long COVID [2]. Studies revealed that 52% of the subjects among the studied population showed fatigue/myalgia post-COVID-19 [3,4]. A survey done by the Office for National Statistics (ONS) in the United Kingdom suggests that about one in five people have symptoms of long COVID five weeks after an initial infection and one in ten after twelve weeks [5]. The chronic phase of COVID-19 is speculated to be perpetual, with impaired functional status and quality of life [6].

Though the data on COVID fatigue is still emerging, viral infections are known to trigger chronic fatigue syndrome (CFS), also known as myalgic encephalomyelitis (ME), in patients. There are no specific biomarkers, and diagnosis is typically based on symptoms. In fact, a subset of patients suffering from COVID-19 satisfied the diagnostic criteria of CFS/ME [7] In addition, major post-acute COVID-19 symptoms resemble post-infectious ME/CFS [8]. The changes in neurotransmitter levels, inflammation, psychological disorders, stress levels, and cognitive dysfunction are thought to be contributing factors in fatigue [2]. An increase in the level of pro-inflammatory cytokines and overexpression of interleukin 6 (IL-6) are associated with persistent inflammation and fatigue [9]. Further, immune dysregulation and mitochondrial dysfunction are common causes of fatigue after viral infection [10]. Thus, management approaches that address these varied patho-physiologies can be evaluated for post COVID fatigue.

While the majority of current treatments for fatigue are palliative, including rehabilitation through spa facilities with multidisciplinary interventions, and are restricted to alleviating symptoms [11], there are indications that certain supplements may be useful in addressing factors potentially involved in the pathogenesis of fatigue. Probiotics have been evaluated in the management of CFS. A significant decrease in anxiety symptoms and modifications in the well-being status, inflammatory and oxidative indexes in CFS patients were seen with probiotics supplementation [12,13]. Antioxidants and immunomodulators have also been explored to combat fatigue [14,15,16]. Lactoferrin and enzymes such as lysozyme, catalase, bromelain and papain are known to function as immunomodulators [17,18,19,20] as well as in combating oxidative stress [21]. Serratiopeptidase is used for its anti-inflammatory and analgesic activity [22]. With this understanding, it is rational to examine the effect of enzymes and probiotics supplementation on COVID-19 induced fatigue.

Early assessments and intervention are critical in reducing COVID-19 induced fatigue, irrespective of initial illness severity [4]. To the best of our knowledge, no interventional study to reduce post-COVID fatigue has been published. In our previously published case series, a 14-day supplementation of enzymes and probiotics (ImmunoSEB and ProbioSEB CSC3) resulted in a significant reduction in fatigue, as measured by the Chalder Fatigue Scale-11 [23]. To further validate these findings, we designed a randomized, multicentric, double blind, placebo-controlled trial to evaluate the efficacy of ImmunoSEB (multi-enzyme formulation of Peptizyme SP, an enteric coated serratiopeptidase, bromelain, amylase, lysozyme, peptidase, catalase, papain, glucoamylase and lactoferrin) and ProbioSEB CSC3 (probiotics blend of Bacillus coagulans LBSC (DSM 17654), Bacillus subtilis PLSSC (ATCC SD 7280) and Bacillus clausii 088AE (MCC 0538)) on COVID-19 induced fatigue.

## 2. Materials and Methods

### 2.1. Materials

The investigational products (IP), ImmunoSEB and ProbioSEB CSC3, were supplied by Specialty Enzymes and Probiotics. The placebo used was maltodextrin. The packaging and labelling for both the IP and placebo were the same, except for the coded batch numbers used for differentiation.

### 2.2. Ethics and Informed Consent

The present clinical trial was conducted as per the ethical principles contained in the current revision of the “Declaration of Helsinki 2013”, ICH harmonized guideline integrated addendum to ICH E6(R1): Guidelines for Good Clinical Practice ICH E6(R2) and following the “Ethical Guidelines for Biomedical Research on Human Subjects” issued by the Indian Council of Medical Research and all other applicable laws and regulations of the country. Informed written consent was obtained from all participants. No vulnerable subject participated in the study. The trial was conducted at three centers in India (Swasthya Hospital, Bhopal, India; Samvedna Hospital, Varanasi, India; and Chirayu Medical College & Hospital, Bhopal, India), by qualified investigators for a duration of 14 days. The trial was registered with the Clinical Trial Registry of India as per Indian regulations with the following registration number: CTRI/2021/05/033576. Date of approval: 10 May 2021.

### 2.3. Selection of Study Subjects

A randomized, multicentric, double blind, 2-Arm parallel design, placebo-controlled clinical trial was conducted on 200 patients that did not have an active SARS-CoV-2 infection, as determined by a negative COVID-19 test, with a complaint of post-COVID fatigue. Patients were required to have a positive COVID-19 test at any time in the past. Patients were recruited from the outpatient department at the three sites.

#### 2.3.1. Inclusion Criteria

Patients who provided written informed consent; males or non-pregnant, non-lactating females aged ≥18 and ≤75 years (both inclusive); RT-PCR confirmed diagnosis of COVID-19 at any time followed by an RT-PCR negative test; patients experiencing fatigue and muscle weakness; able to take the drug orally and comply with study procedures; and women of childbearing potential with a negative urine pregnancy test.

#### 2.3.2. Exclusion Criteria

Patients with severe to critical health condition such as prior known respiratory distress (RR ≥ 30 times/min), finger oxygen saturation ≤90% in a resting state, arterial partial pressure of oxygen (PaO_2_)/concentration of oxygen inhalation (FiO_2_) ≤300 mmHg (1 mmHg = 0.133 kPa), respiratory failure or on mechanical ventilation, in shock, ICU needed for other organ failure; patients with other viral pneumonia; patients unable to take food or drugs due to coma or intestinal obstruction; consumption of other oral probiotic supplements during the trial; patients with severe underlying diseases that affects survival, including uncontrolled malignant tumor with multiple metastases that cannot be resected, blood diseases, dyscrasia, active bleeding, severe malnutrition, etc.; women who are pregnant or lactating, or subjects (including male subjects) having a pregnancy plan (including plans for sperm donation or egg donation) during the study period; patients allergic to systemic enzyme supplements; patients facing imminent death in the opinion of the clinical team; patients with Hb less than 8 mg/dL; and patients who have participated in any other clinical study within 2 weeks prior to randomization were considered ineligible to participate in the study.

### 2.4. Study Design, Randomization and Treatments

**Figure 1:**
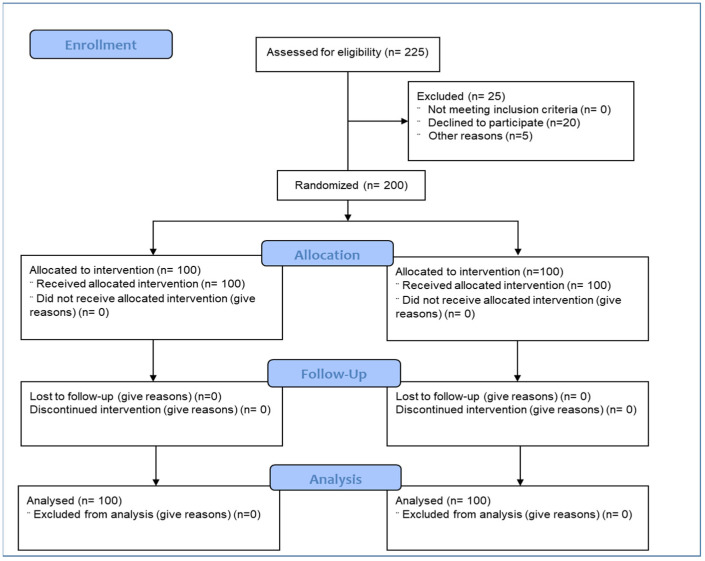
Participant flow chart.

Two hundred patients were randomized (block randomization was done using the online randomization tool www.randomization.com, accessed on 16 August 2021) in a 1:1 ratio to either the test arm (*n* = 100) that received the oral supplements ImmunoSEB (500 mg/capsule) + ProbioSEB CSC3 (5 billion CFUs /capsule) or the control arm (*n* = 100) that received a placebo for 14 days. Patients received four capsules of ImmunoSEB/placebo daily (two capsules in the morning and two in the evening) on an empty stomach (1 h before or 2 h after a meal) with 1–2 cups of warm or room temperature water. They also received 2 capsules of ProbioSEB CSC3/placebo daily, to be taken with lunch. The statistician generated the allocation sequence and concealed envelopes were used for treatment allocation. The Investigators enrolled the participants and assigned the intervention based on the randomization list. The participants, the Investigators and the study team were blinded to the treatment allocation (Figure 1). No changes or amendments were made to the approved protocol after the trial commenced and no interim analysis was done during the study period.

### 2.5. Endpoints: Efficacy and Safety Variables

Primary endpoints were set to study efficacy outcomes like the proportion of patients showing improvement in physical fatigue on CFQ-11 and the proportion of patients showing improvement in mental fatigue on CFQ-11 on day 14. The fatigue assessment was done by using the validated CFQ-11, an 11 item self-report instrument [24,25]. CFQ-11 has two scoring systems, bimodal and Likert. It has a straightforward answering system to measure fatigue or “tiredness” ranging from asymptomatic to maximally symptomatic (‘better than usual’, ‘no worse than usual’, ‘worse than usual’ and ‘much worse than usual’). The bimodal scoring system allows the differentiation of “cases”, i.e., the presence of fatigue vs. “non-cases”, i.e., the absence of fatigue. Briefly, “better than usual”/”no worse than usual” are scored a 0 and “worse than usual”/”much worse than usual” are scored a 1. The sum of all 11 binary scores is calculated and a total score of four or greater meets the criteria for fatigue. The 4-point Likert scoring system weighs the severity of fatigue (better than usual = 0, no more than usual = 1, worse than usual = 2, much worse than usual = 3, for a maximum score of 33), with a higher score indicating greater fatigue. The CFQ-11 comprises two subscales that evaluate fatigue in the physical (questions 1–7, score of 0–21) and mental (questions 8–11, score of 0–12) domains.

In the current study, we used (i) bimodal scoring to determine case-status (fatigue vs. non-fatigued); (ii) Likert scoring to determine severity of fatigue using the total CFQ-11 score; and (iii) Likert scoring for the subscales of physical and mental fatigue. Secondary endpoints include proportion of patients showing improvement on day 4, day 8, day 11, proportion of patients requiring additional therapy for fatigue, and adverse events.

### 2.6. Statistical Analysis

The sample size calculated (assuming effect size = 20%, α = 0.05, enrolment 1:1 and power 80%) was 176. A total of 200 subjects were recruited to cover potential dropouts during the study period. Data were analyzed with 5% significance level (confidence interval 95%) and maintaining a minimum power of 80% for study. The categorical variables were expressed as frequencies and percentages, and continuous variables as mean and standard deviation. The data were analyzed using z test statistics. * *p* < 0.05 was considered statistically significant.

## 3. Results

A total of 200 Asian healthy males and non-pregnant, non-lactating females were randomized to the placebo (*n* = 100) and test arms (*n* = 100). Recruitment was done from May 12, 2021 to May 31, 2021. Last patient last visit including follow up was completed on June 16, 2021. The Principal Investigator and the clinical trial team assessed study compliance at each visit along with the safety and efficacy parameters as per the schedule of events (Table 1). The analysis was performed on the Intention to Treat population, i.e., 100 patients in each arm.

The mean ages of subjects in the control and test arms were 41.29 years (range 20–75) and 41.17 years (range 20–75), respectively. The male to female ratio was 65:35 and 62:38 in the control and test arms, respectively and 17% of subjects in the control arm had comorbidities vs. 12% of subjects in the test arm. The mean days post resolution of SARS-CoV-2 infection was 18.5 and 20.6 days in the control and test arms, respectively. There was no statistically significant difference in age or mean days to enrolment post negative COVID test across study arms (Table 2).

### 3.1. Analysis of Efficacy

**Figure 2:**
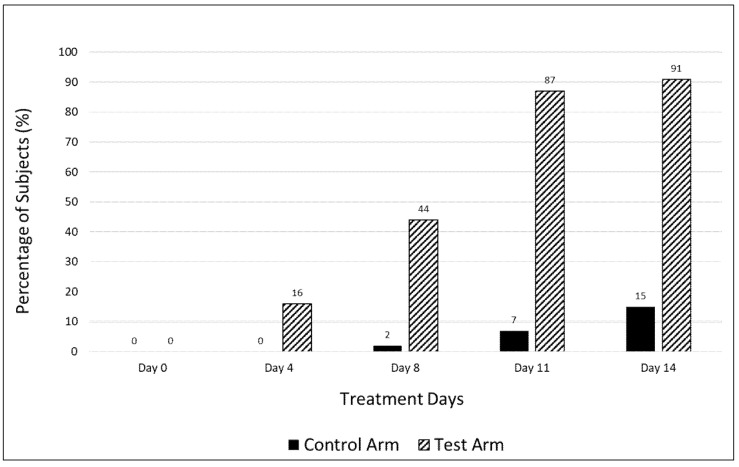
Percentage of fatigue-free subjects in the control and test arms at various time points during the treatment period.

The supplemental treatment resulted in resolution of fatigue in a significantly greater percentage of subjects in the test arm vs. the control arm (91% vs. 15%) on day 14 as assessed by the CFQ-11 bimodal scoring system. Of the 9% of subjects in the treatment arm that were still fatigued on day 14, over half had a lower fatigue score as compared to baseline, though they did not meet the criterion for being fatigue free. A beneficial effect was seen even at earlier time points, with a greater proportion of patients in the test arm being fatigue free on days 4 (16% vs. 0%), 8 (44% vs. 2%), and 11 (87% vs. 7%) vs. the control arm (Figure 2). All patients suffered from fatigue at baseline, as determined by the CFQ-11.

The average total fatigue scores on day 0 were comparable in the control vs. the test arm: 25.69 vs. 25.78. There was a progressive decline in average fatigue scores as determined by the CFQ-11 Likert scoring system in both arms over the two-week treatment period as compared to baseline. Subjects in the test arm showed a significantly greater reduction in total fatigue when compared to the control arm at all time points: control arm vs. test arm day 4, 23.26 vs. 18.72; day 8, 21.75 vs. 14.98; day 11, 20.55 vs. 10.82; and day 14, 19.91 vs. 8.54 (*p* < 0.001).

**Figure 3:**
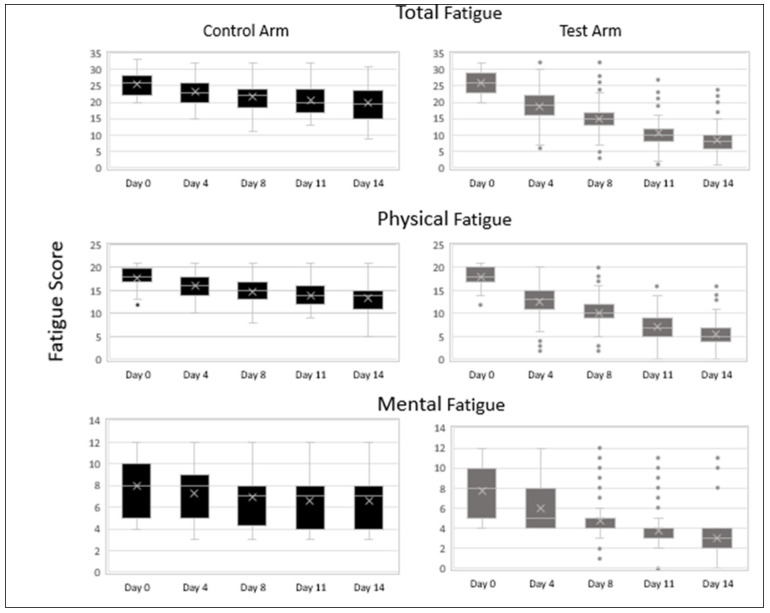
CFQ-11 Fatigue scores (total fatigue, physical fatigue and mental fatigue) of subjects in the control and test arms at various timepoints during the treatment period. Data are represented as median (the line dividing the box), mean (marked with an X), interquartile range (box), range (whisker) and outliers (dots). (*n* = 100).

Analysis by subscales also showed a significantly greater reduction in physical fatigue and mental fatigue in the test arm at all time points when compared to the control arm (*p* < 0.001). There is less variability in the data in the test arm on days 8, 11 and 14 when compared to days 0 and 4 in the test arm as well as when compared to days 8, 11 and 14 in the control arm. The median values of total, physical and mental fatigue are substantially lower on day 4 (19, 13, 5), day 8 (15, 10, 5), day 11 (10, 7, 4) and day 14 (8, 5, 3), respectively, when compared to day 0 (26, 18, 8) in the test arm as well as when compared to day 4 (23, 16, 8), day 8 (22, 15, 7), day 11 (20, 14, 7) and day 14 (19.5, 14, 7) in the control arm (Figure 3). The outliers in the upper range of the test arm represent subjects that were still experiencing fatigue at the end of the 14-day treatment period.

**Figure 4:**
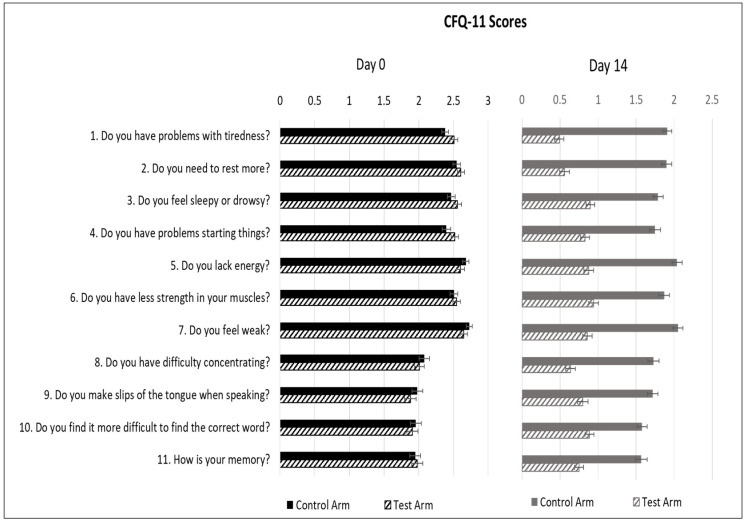
Average CFQ-11 scores of individual measures of fatigue of subjects in the control and test arms on day 0 and day 14 of the treatment period All data are presented as mean ± SEM (*n* = 200).

Average fatigue scores for individual questions in the control arm vs. test arm were comparable on day 0: Question 1, 2.38 vs. 2.51 (*p* = 0.08); Q2, 2.55 vs. 2.61 (*p* = 0.42); Q3, 2.47 vs. 2.56 (*p* = 0.26); Q4, 2.40 vs. 2.52 (*p* = 0.13); Q5, 2.68 vs. 2.60 (*p* = 0.28); Q6, 2.51 vs. 2.55 (*p* = 0.61); Q7, 2.73 vs. 2.65 (*p* = 0.24); Q8, 2.08 vs. 2.01 (*p* = 0.50); Q9, 1.98 vs. 1.88 (*p* = 0.37); Q10, 1.96 vs. 1.91 (*p* = 0.65); and Q11, 1.95 vs. 1.98 (*p* = 0.79). On day 14, there was a significant reduction in all individual measures of physical fatigue (tiredness, need to rest, drowsiness, ability to do things, energy level, muscle strength and feeling of weakness) as well as mental fatigue (concentration, focus and memory) in the test arm vs. the control arm. Question 1, 1.92 vs. 0.49; Q2, 1.90 vs. 0.57; Q3, 1.80 vs. 0.90; Q4, 1.76 vs. 0.82; Q5, 2.04 vs. 0.88; Q6, 1.87 vs. 0.94; Q7, 2.05 vs. 0.87; Q8, 1.74 vs. 0.65; Q9, 1.73 vs. 0.80; Q10, 1.58 vs. 0.89; Q11, 1.58 vs. 0.76 (*p* < 0.001) (Figure 4). As seen in the figure, on day 14, all subjects in the test arm had fatigue scores < 1, indicating “better than usual” status on all parameters of physical and mental fatigue.

### 3.2. Analysis of Safety

Vitals (pulse rate, respiratory rate, blood pressure, oxygen saturation and body temperature) were collected for all patients at baseline, at specific time points during the study and at the end of study and were in the normal range. No subject in either arm reported any adverse event(s) including nausea, vomiting or diarrhea at any time during the study, suggesting the safety and tolerability of supplementation with ImmunoSEB + ProbioSEB CSC3. Compliance with product intake was 100% and no subject reported having to skip any dose or stop supplement intake due to an adverse reaction.

## 4. Discussion

The current study represents, to our knowledge, the first report of a randomized controlled trial demonstrating the efficacy of dietary supplements in resolving fatigue, following SARS-CoV-2 infection. This is important as there is considerable concern that COVID-19 disease triggers post-viral fatigue syndromes [26,27,28,29]. During follow-up in survivors of other coronaviruses, such as severe acute respiratory syndrome (SARS), 64% reported fatigue at 3 months, 54% at 6 months and 60% at 12 months [30,31]. Following Middle East respiratory syndrome (MERS), 48% had clinically relevant fatigue after 12 months [32]. A lengthy post infection fatigue burden impairs quality of life and will have a significant impact on individuals, employers and healthcare systems, if not managed effectively.

We evaluated the effect of the supplements ImmunoSEB and ProbioSEB CSC3 on the resolution of post-COVID fatigue using CFQ-11. The bimodal scoring system allows the differentiation of “cases” vs. “non-cases”. This method for “caseness” is validated and closely resembles other fatigue questionnaires [33,34,35,36]. In the test arm, 91% of subjects were fatigue-free at end of treatment (EOT, day 14). Of the 9% of subjects in the treatment arm that were still fatigued on day 14, over half had a lower fatigue score as compared to baseline, though they did not meet the criterion for being fatigue free. These subjects may need a longer period of supplementation to completely resolve their fatigue. It is worthwhile to note that subjects in the test arm were recruited from between 4–87 days post their acute COVID infection, suggesting the efficacy of our supplemental regimen in cases of early fatigue as well as long term fatigue.

Significant improvement in fatigue severity, as evaluated using the Likert scoring system, was seen on all days tested, as compared to the baseline. The reduction in total fatigue scores was significantly greater in the test arm as compared to the control arm on all days tested, thus showing proof of efficacy of the supplemental regimen in reducing the severity of post COVID fatigue. Analysis by subscales demonstrated that the supplements were effective in reducing both physical and mental fatigue. The individual measures of physical fatigue include tiredness, need to rest, drowsiness, ability to do things, energy level, muscle strength and feeling of weakness. The mental fatigue subscale measures symptoms such as difficulty concentrating, ability to focus or think clearly and memory difficulties, all of which have been described as “Brain Fog”. In the test arm, all parameters had a score of <1 indicating “better than usual” status on day 14, suggesting the efficacy of our supplemental regimen in addressing a wide array of post COVID-19 symptoms.

The post-acute recovery phase of COVID- 19 is assumed to be accompanied by oxidative stress and inflammation which causes physical and mental fatigue [37]. A literature review on chronic fatigue syndrome found that many patients have persistent low-level inflammation, possibly triggered by infection [38]. Immune dysfunction, oxidative stress and inflammation have been observed in patients with fatigue, and account for a number of fatigue symptoms. Inflammatory changes in the brain cause “brain fog” which includes symptoms like memory loss and trouble concentrating. Gut dysbiosis has also been linked with chronic fatigue [39,40]. Antivirals, antioxidants, immunosuppressive agents and nutrients that support mitochondrial function have been explored individually in the management of fatigue [37]. The heterogeneity of fatigue makes it difficult to find one solution that provides 100% benefit for all patients [10]. Thus, we have explored the efficacy of a combination of dietary supplements comprising systemic enzymes (ImmunoSEB, a multi-enzyme formulation of Peptizyme SP, an enteric coated serratiopeptidase, bromelain, amylase, lysozyme, peptidase, catalase, papain, glucoamylase and lactoferrin) and probiotics (ProbioSEB CSC3, a blend of *Bacillus coagulans* LBSC (DSM 17654), *Bacillus subtilis* PLSSC (ATCC SD 7280) and *Bacillus clausii* 088AE (MCC 0538)) in the management of post COVID fatigue.

The anti-inflammatory, anti-oxidant and immunomodulatory properties of systemic enzymes are previously documented in the literature [17,19,41,42,43] and likely effected the reduction of COVID-19 fatigue observed in the present trial: systemic enzymes are useful as immunotherapeutics as they can modulate the local availability of immunostimulatory and immunosuppressive signals [44]; serratiopeptidase and bromelain possess anti-inflammatory activity [22,41,42]; catalase and lysozyme reduce oxidative stress [21,43]; serratiopeptidase, lysozyme, lactoferrin and bromelain have anti-viral activity [45,46,47]; and papain, bromelain, lysozyme and lactoferrin have immunomodulatory effects [17,18,19]. Specifically, the potential of bromelain in inhibiting SARS-CoV-2 infection [46] and catalase in reducing oxidative stress in SARS-CoV-2 infection [48] has been demonstrated in the reported studies.

In addition to their immunomodulatory [49], anti-inflammatory [50], antioxidant [51] and antiviral [52] actions, probiotics have been shown to boost mood, improve cognitive function and reduce fatigue: probiotics improve well-being as well as inflammatory and oxidative indexes in CFS/ME patients [13]; probiotics regulate brain health via the gut–brain axis [53]; probiotic supplementation significantly improved mood and sleep quality and reduced depression, anger and fatigue [54,55,56]. This evidence suggests that probiotics have the potential to improve measures of both physical and mental fatigue, as demonstrated in the current trial.

The results obtained in this randomized, placebo-controlled trial are in good agreement with our previously reported case series on post-COVID fatigue [23], and further build on our previous in vitro and clinical data on these dietary supplements: In an open-label clinical trial in patients hospitalized with mild to moderate COVID-19 disease, supplementation with ImmunoSEB and ProbioSEB CSC3 resulted in earlier clinical improvement and faster reduction in CRP levels [57]. In our in vitro study, ImmunoSEB showed antiviral activity against the SARS-CoV-2 virus [47].

Our study has a few limitations worthy of discussion. Our study only assessed participants during a 2-week period. However, this may be sufficient as a majority of the subjects in the test arm showed a resolution of fatigue at this time point. We did not specifically collect information about depression and psychiatric history at baseline; thus, this was not adjusted as a covariate. However, no patient reported having a psychiatric illness as part of their medical history. As mentioned, we reported post-COVID fatigue at an early timepoint (an average of 3 weeks post resolution of infection). We would recommend that studies are designed to evaluate the effectiveness of this supplemental therapy in patients with persistence of fatigue six months or beyond to address the needs of patients suffering from chronic fatigue. The treatment period in our study was a one-time intervention of 14 days with no long-term follow up. It would be worthwhile to conduct a long-term follow up of patients in future studies to evaluate potential recurrence of fatigue. Testing for certain inflammatory and immunity markers in these patients may provide further insight into the mechanism of action of the current supplemental regimen.

## 5. Conclusions

This study demonstrates that a 14 days supplementation of ImmunoSEB + ProbioSEB CSC3 resolves post-COVID-19 fatigue. The proposed supplement regimen significantly reduces the burden of both, physical and mental fatigue and is effective as an early intervention in the recovery of COVID-19 patients, many of whom continue to experience severe fatigue including muscle weakness and “brain fog” several months after initial infection. Thus, while researchers are still characterizing post-COVID sequelae, we suggest the addition of these dietary supplements to other evidence-based multidisciplinary care approaches to improve functional status and quality of life in these patients.

## Figures and Tables

**Table 1:** Schedule of assessments.

Parameter	Screening	Treatment														EOT
Visits	1	2														3
Day (±days)	24–48 h	1	2	3	4	5	6	7	8	9	10	11	12	13	14	15
Written Informed Consent	X															
Inclusion/Exclusion Criteria	X															
Medical and surgical History	X															
Physical Examination	X															
Vital signs	X				X				X			X				X
Demographic Information	X															
IP administration		X	X	X	X	X	X	X	X	X	X	X	X	X	X	
Urine pregnancy test (In case of a female subject)	X															
Chalder Fatigue Scale	X				X				X			X			X	X ^1^
Patient Diary		X	X	X	X	X	X	X	X	X	X	X	X	X	X	
Adverse events		X	X	X	X	X	X	X	X	X	X	X	X	X	X	X
Concomitant Medications		X	X	X	X	X	X	X	X	X	X	X	X	X	X	X

^1^ Collection of Completed Questionnaires.

**Table 2:** Demographics and baseline characteristics.

	Control Arm	Test Arm	* *p* Value
Age (years); SD; Range	41.29 ± 13.0 (20–75)	41.17 ± 12.9 (20–75)	0.11
Males: Females (%)	65:35	62:38	-
Subjects with co-morbidities (%)	17	12	-
Days to enrolment post negative COVID test; Range	18.5 (2–48)	20.6 (4–87)	0.20

* *p* values were calculated using z test statistics.

## Data Availability

The conditions of our ethics approval do not permit public archiving of the data supporting the conclusions of the study. However, data described in the manuscript, code book, and analytic code will be made available upon request.
